# Hyperparasitism in bat flies (Diptera: Streblidae): new records and interaction networks in the Neotropics

**DOI:** 10.1007/s00436-024-08221-1

**Published:** 2024-06-26

**Authors:** Camila López-Rivera, Laura Natalia Robayo-Sánchez, Alejandro Ramírez-Hernández, Jesús Alfredo Cortés-Vecino, Jerson Andrés Cuéllar-Sáenz, Juan Diego Villar, Fredy Arvey Rivera-Páez, Paula Andrea Ossa-López, José Jaime Henao-Osorio, Alexandra Cardona-Giraldo, Erika Mayerly Ospina-Pérez, Marylin Hidalgo, Héctor E. Ramírez-Chaves

**Affiliations:** 1 Maestría en Ciencias Biológicas, Facultad de Ciencias Exactas y Naturales, Universidad de Caldas, Calle 65 No. 26-10, 170004 Manizales, Caldas, Colombia; 2 Grupo de Investigación en Genética, Biodiversidad y Manejo de Ecosistemas (GEBIOME), Departamento de Ciencias Biológicas, Facultad de Ciencias Exactas y Naturales, Universidad de Caldas, Calle 65 No. 26-10, 170004 Manizales, Caldas, Colombia; 3 Grupo de Investigación Parasitología Veterinaria, Laboratorio de Parasitología Veterinaria, Universidad Nacional de Colombia, Bogotá, D.C Colombia; 4 Grupo Enfermedades Infecciosas, Departamento de Microbiología, Pontificia Universidad Javeriana, Bogotá, D.C Colombia; 5 Universidad de La Salle, Bogotá, D. C Colombia; 6 Centro de Museos, Museo de Historia Natural, Universidad de Caldas, Calle 58 No. 21-50, 170004 Manizales, Caldas, Colombia

**Keywords:** Chiroptera, Ecological interactions, Fungi, Trombidiformes, Streblidae

## Abstract

Hyperparasitism is defined as the interaction where one parasite is infected by another parasite. In bat flies (Streblidae and Nycteribiidae), both hyperparasites and microparasites (bacteria, viruses, fungi, and arthropods such as mites) have been documented. Fungi belonging to the order Laboulbeniales are microscopic parasites of a wide diversity of arthropod hosts. Three genera exclusively target bat flies: *Arthrorhynchus*, which parasitizes species within Nycteribiidae in the Eastern Hemisphere, while genus *Gloeandromyces* and *Nycteromyces* parasitize Streblidae in the Western Hemisphere. Among the hyperparasitic arthropods, mites of family Neothrombidiidae, particularly the monospecific genus *Monunguis*, are known to parasitize bat flies. Here we present the first records of the hyperparasites *Monunguis streblida* and *Gloeandromyces pageanus* f. *polymorphus* parasitizing Streblidae bat flies in Colombia and a summary of these hyperparasitic interactions in the Neotropics. We detected fungi and mites parasitizing bat flies that were collected in the Magdalena River Basin, Colombia, in field expeditions in 2018, 2022, and 2023. We identified 17 bat flies and two species of hyperparasites, specifically *M. streblida* and the fungi *Gloeandromyces*. Our search for reports of these interactions in the Neotropics revealed that seven species of *Trichobius* (Streblidae) are parasitized by *M. streblida*, whereas *Paratrichobius longicrus* (Streblidae) is parasitized by *Gloeandromyces pageanus f. polymorphus*. These interactions have been reported in 11 countries, but our records are the first of *M. streblida* and Laboulbeniales fungi parasitizing bat flies in Colombia. So far, a total of 14 species of fungi and one species of mite have been associated with 19 species of bat flies, which in turn, are linked to 15 species of Neotropical bats.

**Supplementary Information:**

The online version contains supplementary material available at 10.1007/s00436-024-08221-1.

## Introduction

Hyperparasitism is defined as an interaction in which one parasite is infected by another parasite (Sullivan and Völkl 1999). This interaction might be common in nature, indirectly affecting the fitness of the main host by playing an important role in regulating host-parasite cycles, shaping disease dynamics and other ecological connections, including their evolution (Parratt and Laine 2016). Macroparasites and microparasites have been reported in bat flies (Streblidae) and include bacteria (*Bartonella* Strong et al. 1915 emend. Birtles et al. 1995), fungi (Laboulbeniaceae), other arthropods (mites), blood parasites (*Polychromophilus* Dionisi, 1901, *Trypanosoma* Gruby, 1843, and *Wolbachia* Hertig, 1936), and, to a lesser extent, viruses (Anderson and May 1981; Blackwell et al. 2020; Lee et al. 2021). These associations have been reported mainly in Europe, North America, and Africa and less frequently in Asia, Oceania, and South America (Szentiványi et al. 2019).

Fungi of the order Laboulbeniales (Ascomycota: Laboulbeniomycetes) are microscopic hyperparasites of a great diversity of arthropod hosts (Blackwell et al. 2020). Laboulbeniales are usually host-specific; about 80% of the described species of this order are found on Coleoptera, 10% on Diptera (Weir and Hammond 1997), and the remaining 10% are divided into many other taxa such as Arachnida, Diplopoda, and Hexapoda (Weir and Hammond 1997; Haelewaters et al. 2017b; Santamaria et al. 2017; Walker et al. 2018). The Laboulbenial fungi associated with Diptera belong to eight genera, three of which are exclusive to bat flies (Hippoboscoidea): *Arthrorhynchus* Kolen, 1857, *Gloeandromyces* Thaxter, 1931, and *Nycteromyces* Thaxter, 1917 (Walker et al. 2018; Szentiványi et al. 2019). Globally, the laboulbenial fungi are represented in the Eastern Hemisphere by species of *Arthrorhynchus* parasiting bat flies within Nycteribiidae and in the Western Hemisphere represented by taxa of *Gloeandromyces* and *Nycteromyces* parasitizing bat flies within Streblidae (Haelewaters et al. 2017b; Szentiványi et al. 2019). Thaxter (1917, 1931) first reported the genera *Gloendromyces* and *Nycteromyces* in two Neotropical countries (Grenada and Venezuela). Since then, the records of these genera have been documented in seven countries in America (Bertola et al. 2005; Fritz 1983; Haelewaters et al. 2017b, 2018b).

Among arthropod hyperparasites, mites of the family Neothrombidiidae (Acari: Trombidiformes) are parasites of Arachnida, Coleoptera, Diptera, and Hemiptera, among others (Mąkol and Wohltmann 2012). Neothrombidiidae are distributed almost worldwide (Beron 2007). However, only four genera have been described: *Anomalothrombium* André, 1936; *Discotrombidium* Feider, 1977; *Monunguis* Wharton, 1938; and *Neotrombidium* Leonardi, 1901*.* Specifically, the monospecific genus *Monunguis* that parasites bat flies is represented by *Monunguis streblida* Wharton, 1938, which was only described based on the larval stages and the adults are unknown (Lindquist and Vercammen-Grandjean 1971). *Monunguis streblida* has been documented parasitizing mainly three species of bat flies (Streblidae): *Megistopoda aranea* (Coquillett, 1899); *Trichobius pseudotruncatus* Jobling, 1939; and *Trichobius dugesii* Townsend, 1891, probably collected from bat species such as *Artibeus jamaicensis* Leach, 1821, *Glossophaga soricina* (Pallas, 1766) (Chiroptera, Phyllostomidae), and *Myotis nigricans* (Schinz, 1821) (Vespertilionidae) (Lindquist and Vercammen-Grandjean 1971).

*Monunguis streblida* was previously considered restricted to Mexico, specifically to the “Cinquo de Mayas” cave in the state of Yucatán, and to the Dominican Island (Borland 1956; Lindquist and Vercammen-Grandjean 1971). Recently, da Silva Reis et al. (2019) reported the presence of *M. streblida o*n streblid bat flies of the *Trichobius dugesii* complex; *Anastrebla caudiferae* Wenzel, 1976; and *A. modestini* Wenzel, 1966, collected from the host bat *Anoura geoffroyi* Gray, 1838, in Brazil, specifically in the State of the Minas Gerais. These records extended *M. streblida* distribution more than 5000 km south, confirming its presence in South America. Hence, currently, the presence of *M. streblida* is only known in Mexico and Brazil. Here, we present the first records of hyperparasitism on bat flies in Colombia, and we provide a review of reports of hyperparasites involving fungi and mites associated with bat-flies and analyze their interactions in the Neotropics.

## Material and methods

**Fig. 1:**
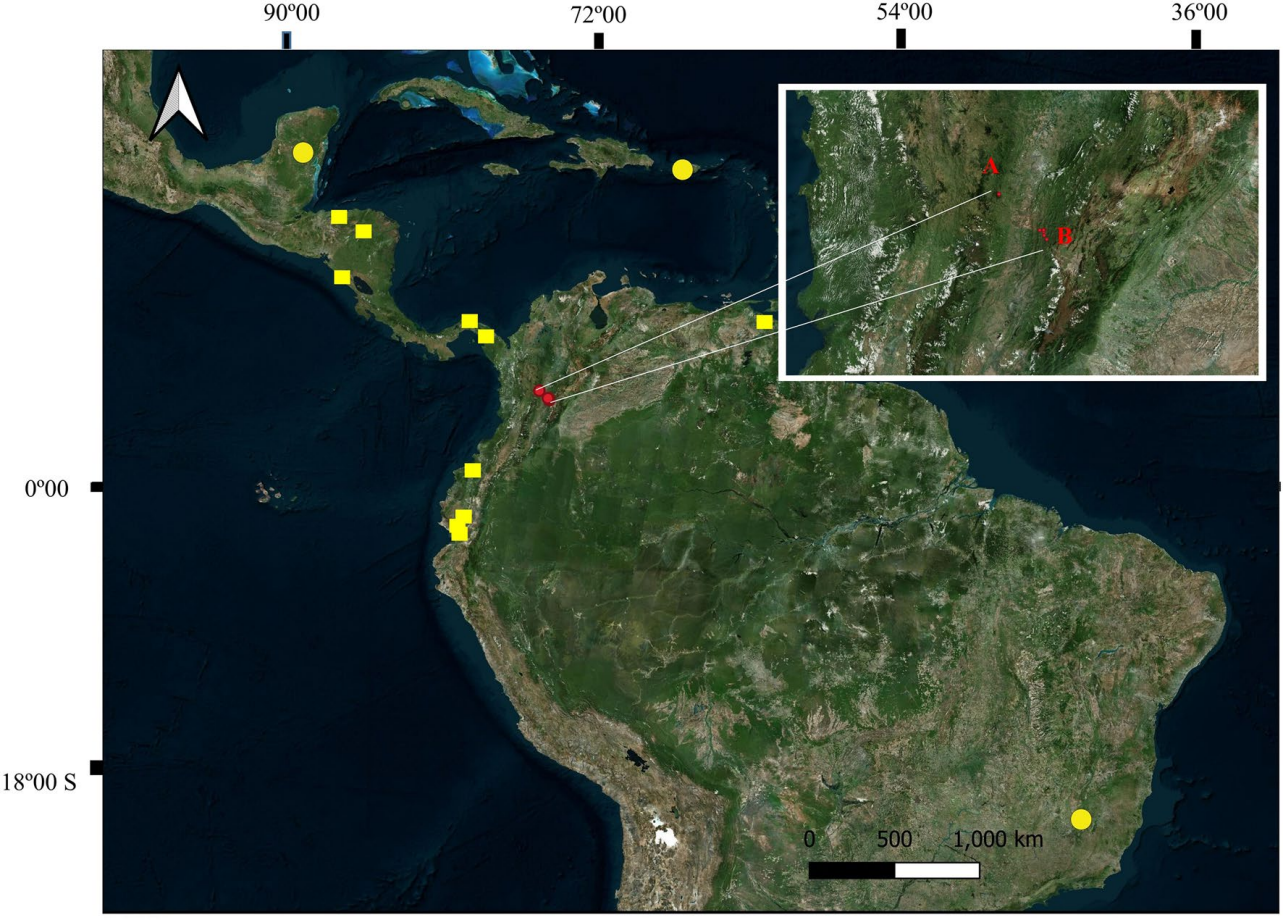
Localities sampled within the Magdalena River Basin in Colombia. Red points denote the localities in which hyperparasites were detected in (**A**) Samaná, Caldas and (**B**) Villeta, Cundinamarca. Yellow circles denote previous cases of hyperparasite mites and yellow squares of hyperparasite Laboulbeniales in the Neotropics

We captured bats and bat flies during April 2018 in Samaná (Department of Caldas) and July 2022, January, and March 2023 in Villeta (Department of Cundinamarca), in the inter-Andean basin of the Magdalena River in Colombia (Fig. 1; Supplementary Table 1). This basin covers 257,438 km^2^ of the Colombian territory, crosses 13 departments, with high anthropogenic influence (Restrepo and Syvitski 2006). The captured bats were placed individually in cotton bags. From each captured bat, we manually collected the flies using entomological forceps and placed in them in Eppendorf tubes with 70% alcohol. For the identification of bats and bat flies, we used taxonomic keys (e.g., Wenzel et al. 1966; Wenzel 1976; Díaz et al. 2021). We deposited the collected specimens at the Museo de Historia Natural of the Universidad of Caldas (MHN-UCa) in Manizales, Colombia. During the identification of the ectoparasitic flies we detected the presence of mites and fungi parasitizing bat flies. The mite specimens were identified using taxonomic keys and species descriptions by Walter et al. (2009) and Lindquist and Vercammen-Grandjean (1971), and for Laboulbeniales fungi, the taxonomic keys of Thaxter (1917, 1931) and Haelewaters et al. (2017b) and the morphological descriptions of Haelewaters and Pfister (2019) and Liu et al. (2020). The collections were executed within the framework permit granted by the National Environmental Licensing Authority (ANLA) to the Universidad de Caldas as stipulated in resolution No. 02497 of December 31, 2018.

### Tripartite interaction network between bat-bat fly-hyperparasite

To represent bat-bat fly-hyperparasite interactions, we used our records and searched for data on the occurrence of Neotropical hyperparasites of bat flies (Laboulbeniales fungi and mites). The last search was performed in August 2023 in Scopus and Web of Science, using keywords such as “hyperparasitism,” “Streblidae,” “fungi,” and “mites.” To construct the tripartite interaction network, we incorporated information regarding the hyperparasite, the bat fly, and the bat involved in each interaction. The network is built by the interaction of two adjacency matrices: the first matrix (web1) represents the interactions observed between bat flies (columns) and bats (rows), and the second matrix (web2) represents the hyperparasite records in each bat flies species. To visualize the network, we used the “plotwed2” function from the “bipartite” package in R that joins both matrices. Hyperparasite, bat flies, and bats are represented as nodes, while the number of record associations among these species is depicted by lines. The thickness of the lines reflects the number of recorded associations (Dorman et al. 2009).

## Results and discussion

We captured 176 bats of the families Phyllostomidae, Vespertilionidae, and Molossidae of which 88 bats were parasitized by 215 Streblidae bat flies belonging to 15 species (Supplementary Table 2 and Fig. S1). Of the 215 bat flies, we found seven *Trichobius joblingi* flies parasitized by the mite *M. streblida* and one *Paratrichobius longicrus* parasitized by a Laboulbeniales fungi (prevalence = 9.3% and 7.69%, respectively, for each bat fly species).

**Fig. 2:**
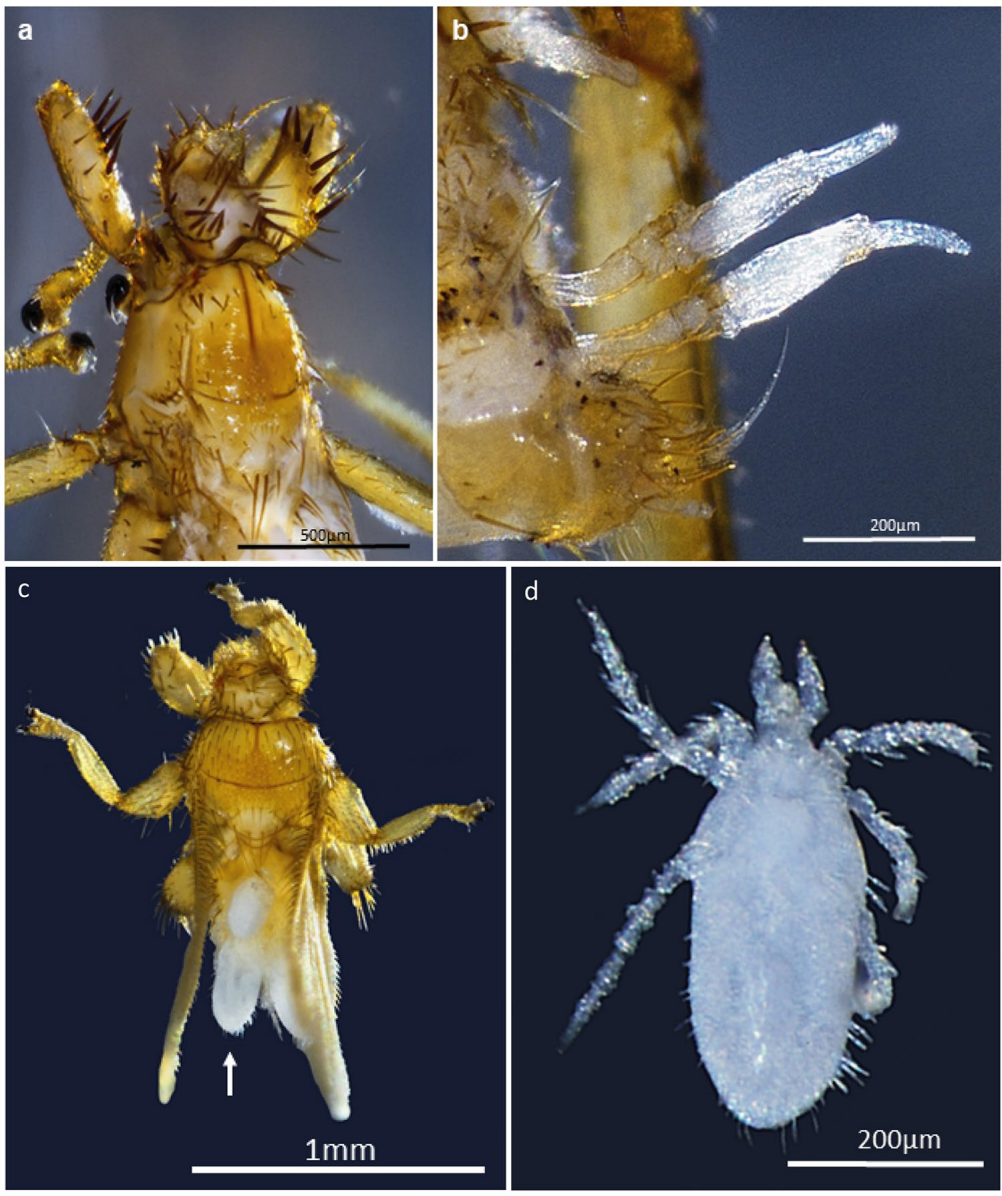
**a** *Paratrichobius longicrus*. **b** Mature thallus of *Gloeandromyces pageanus f. polymorphus*. **c** *Trichobius joblingi*. **d** *Monuguis streblida*

The flies parasitized by the mite *M. streblida* were collected from five host bats of *Carollia perspicillata* (Linnaeus, 1758). The flies were parasitized by two or three mites positioned on the back of their abdomen (Fig. 2). We identified the *M. streblida* specimens by the presence of six segments in its three pairs of legs; the tarsus of the foreleg has a bare parasubterminalia and for having the hysterosome for more than 200 attenuated setae. For *M. streblida*, this is the first record for Colombia and the second for South America with previous records in Brazil (da Silva Reis et al. 2019). We highlight the specific relationship of the larva of *M. streblida* mainly with bat flies *Trichobius* (Lindquist and Vercammen-Grandjean 1971; da Silva Reis et al. 2019). Previous works documented the association of *M. streblida* with bat flies collected from three Phyllostomid bats: *Anoura geoffroyi*, *Artibeus jamaicensis*, and *Glossophaga soricina* (Lindquist and Vercammen-Grandjean 1971; da Silva Reis et al. 2019). In our case, the ectoparasitic flies were collected from *Carollia perspicillata*; therefore, a new host bat is reported for this type of interactions. *M. streblida* usually is attached to the abdomen of its hosts and might prefer flies with developed wings than to flies with rudimentary wings (da Silva Reis et al. 2019). Winged hosts may offer some protection to the mite during its development or greater dispersal capacity (da Silva Reis et al. 2019). In contrast, Lindquist and Vercammen-Grandjean (1971) found larva of *M. streblida* parasitizing bat flies with rudimentary wings, challenging the hypothesis of preference of this mite for species of ectoparasitic flies with developed wings. More studies are still needed to confirm the preference of mites for either flies with fully developed or rudimentary wings.

We found that *Paratrichobius longicrus*, collected from a greater broad-nosed bat *Platyrrhinus vittatus* (Peters, 1860), was the only bat fly parasitized by *Gloeandromyces pageanus f. polymorphus* Haelew, 2019 (Fig. 2). We found the mature stems of this fungus on the ventral area of the abdomen, near the gonopodium of the bat fly, joining the exoskeleton and forming a thallus. The fungus is recognized by its morphology, with the perithecial venter ending in four conspicuous bumps; it has thallus faintly yellowish, with distinctly darker upper half of cell III, cell I longer than broad, and curved towards posterior side, broadening upwards (Haelewaters and Pfister 2019). Our record of this interaction is the first in Colombia and the fifth in South America where it was previously documented in Venezuela (Thaxter 1917), Brazil (Bertola et al. 2005), Ecuador, and Trinidad and Tobago (Haelewaters et al. 2018a). *Gloeandromyces pageanus f. polymorphus* has been reported on the flies *Trichobius joblingi* and on *T. dugesioides* associated with the bats *C. perspicillata* and *Trachops cirrhosus* (Spix, 1823), respectively (Haelewaters and Pfister 2019). The presence of the fungus near the bat fly gonopodium in our study may be explained by the fact that Laboulbeniales fungi are mainly transmitted during mating of bat flies (Haelewaters et al. 2018b), although other studies (Haelewaters et al. 2017a, 2017b; Walker et al. 2018; Haelewaters and Pfister 2019) show that thalli of different fungal species or morphotypes may be restricted to a particular area of the host body (e.g., *G. pageanus f. alarum*, restricted to the base of the wings).

**Fig. 3:**
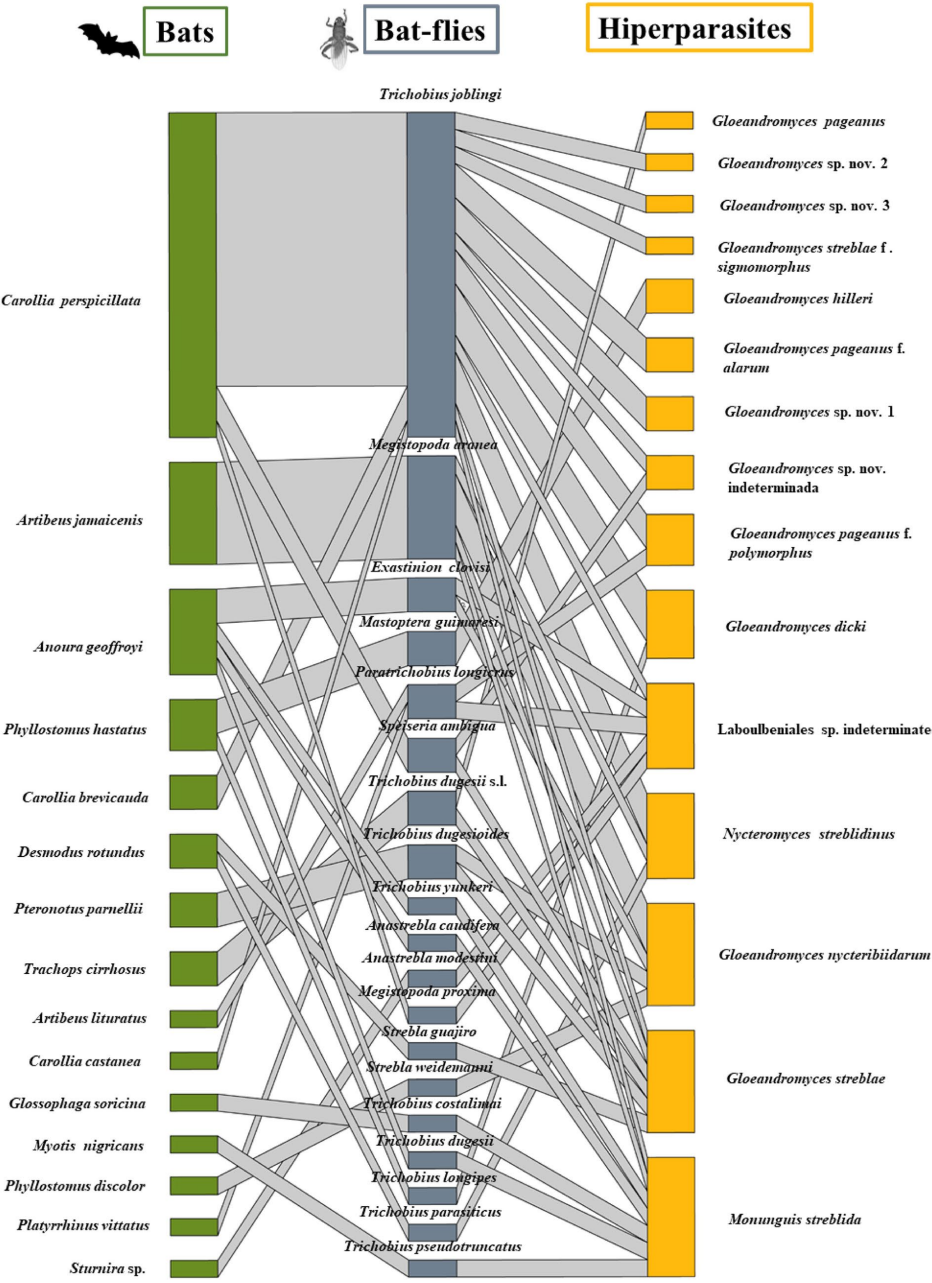
Host-parasite-hyperparasite interaction network. Green nodes represent bat species, gray nodes represent fly species, and yellow nodes represent fungal and mite species. The width of the bar represents the number of association records for each species and the thickness of lines the number of associations reported among pairs of species within the network

The search of hyperparasites of bat flies in the Neotropics showed that this type of interaction has been reported in 11 countries (Supplementary Table 3). A total of 14 species of Laboulbeniales and one species of mite were found in 19 bat fly species (Streblidae), associated with 15 species of bats of the families Mormoopidae, Phyllostomidae, and Vespertilionidae, including our occurrence data (Fig. 3). Laboulbeniales species belonged to the genera *Gloeandromy*ces and *Nycteromyces*, parasitizing 14 of the 19 species of ectoparasitic flies found in our literature review (9 documents reviewed; Supplementary Table 3). The most frequent species was *Gloeandromyces streblae* Thaxter, 1931, interacting with six species of ectoparasitic flies. Furthermore, eight of the 14 Laboulbeniales species had a single host. *Trichobius joblingi* is the bat fly species with the highest number of interactions with Laboulbeniales fungi and with most interactions with bats of the genus *Carollia*. Similarly, according to the literature (Supplementary Table 3), the mite *M. streblida* was found interacting with six species of bat flies, being the only hyperparasite for at least five of them (*Anastrebla caudiferae*, *A. modestini*, *Trichobius dugesii*, *T. dugesii* complex, *T. pseudotruncatus*).

Our review of additional cases also highlights the significant gap in information concerning the ecology and distribution of hyperparasites, such as Laboulbeniales and mites, associated with bat flies in the Neotropics. Over the past two decades, only five studies have addressed this topic (Haelewaters et al. 2017b, 2018b; Walker et al. 2018; Haelewaters and Pfister 2019; Liu et al. 2020). However, hyperparasites of bats have garnered increased ecological attention in recent years (Haelewaters et al. 2018b; Walker et al. 2018). Additionally, the existing data are often fragmented and predominantly derived from a single locality within each country. This limited dataset hinders a comprehensive understanding of the intricate interactions between bats, bat flies, hyperparasites, and their distribution across the Neotropics. Further research is essential to gain a deeper understanding of the transmission mechanisms and the effects of these hyperparasites on both the bat fly and the bat host (Haelewaters et al. 2017b, 2018b). In the case of Laboulbeniales, many of these species are cryptic and challenging to distinguish based on morphology alone and the application of molecular approaches to clarify their taxonomic status is recommended (Haelewaters et al. 2017b).

### Supplementary Information

Below is the link to the electronic supplementary material.

Supplementary file1 (DOCX 991 KB)

## Data Availability

No datasets were generated or analysed during the current study.
