# Supplementary material for: Hyperparasitism in bat flies (Diptera: Streblidae): new records and interaction networks in the Neotropics
Source: Parasitol Res. 2024 Jun 26;123(6):255. doi: 10.1007/s00436-024-08221-1 (PMC11208258; doi:10.1007/s00436-024-08221-1)
Supplement: Supplementary file 1 — Supplementary file1 (DOCX 991 KB) [file 436_2024_8221_MOESM1_ESM.docx]

**Supplementary Table 1** Localities where the field trips were carried out, the asterisk (*) indicates the localities where records of hyperparasites were obtained.

| **Number** | **Municipality** | **Localities** | **Coordinates** | **Elevation m a.s.l** | **Date** |
| --- | --- | --- | --- | --- | --- |
| 1 | Samaná, Caldas | National Natural Park (PNN) Selva de Florencia, Microcuenca Chupaderos, Vereda San Lucas | 05°29'55.7"N,75°02'56.5"W | 1309 | 21/04/2018 |
| 2 | Samaná, Caldas | PNN Selva de Florencia, Microcuenca Chupaderos 1, Vereda San Lucas * | 05°29'28.5"N, 75°02'45.6"W | 1423 | 22/04/2018 |
| 3 | Samaná, Caldas | PNN Selva de Florencia, Microcuenca Chupaderos, Vereda San Lucas | 05°29'40.3"N, 75°02'43.4"W | 1438 | 23/04/2018 |
| 4 | Villeta, Cundinamarca | Salitre Blanco, El Retiro * | 5°02'51"N 74°29'15"W | 1311 | 12/07/2022 |
| 5 | Villeta, Cundinamarca | Salitre Blanco, El Retiro * | 5°02'58"N 74°29'18"W | 1397 | 13/07/2022 |
| 6 | Villeta, Cundinamarca | Salitre Blanco, El Retiro | 5°02'55.3"N, 74°29'131.6"W | 1311 | 15/07/2022 |
| 7 | Villeta, Cundinamarca | Cune, Reserva Forestal La Playita | 5°01'55"N 74°30'02"W | 1042 | 17/07/2022 |
| 8 | Villeta, Cundinamarca | Cune, Chamorro | 05°01'57.3"N, 74°29'34.1"W | 1044 | 12/01/2023 |
| 9 | Villeta, Cundinamarca | Mave, Túnel | 04°56'25.7"N, 74°27'34"W | 1289 | 15/01/2023 |
| 10 | Villeta, Cundinamarca | Salitre Blanco, El Retiro | 05°03'2.3"N, 74°29'28.2"W | 1324 | 16/01/2023 |
| 11 | Villeta, Cundinamarca | Cune, Choquenzá | 05°03'11.3"N, 74°29'37.5"W | 1271 | 17/01/2023 |
| 12 | Villeta, Cundinamarca | Bagazal, Estación del Tren | 04°59'16.4"N, 74°29'22.9"W | 868 | 18/01/2023 |
| 13 | Villeta, Cundinamarca | La Esmeralda, La Esmeralda | 05°03'18.4"N, 74°32'37.9"W | 1999 | 15/03/2023 |

**Supplementary Table 2** Bat fly species found in Samaná, Caldas and Villeta, Cundinamarca. Localities are based on Table 1. Ectoparasite (Ec) and Mammals (M) collections of the Museo de Historia Natural of the Universidad of Caldas (MHN-UCa)

| **Diptera** | **n** | **Chiroptera** | **n** | **Locality** | **Voucher (MHN-UCa)** |
| --- | --- | --- | --- | --- | --- |
| **Streblidae** |  | **Phyllostomidae** |  |  |  |
| *Anastrebla modestini* Wenzel, 1966 | 2 | *Anoura luismanueli* Molinari, 1994 | 1 | 13 | Ec-1794, M-4428 |
| *Aspidoptera delatorrei* Wenzel, 1966 | 8 | *Sturnira giannae* Velazco and Patterson, 2019 | 3 | 4, 5, 6 | Ec-1622,1651, 1764; M-4097, 4098, 4353 |
| *Aspidoptera phyllostomatis* (Perty, 1833) | 1 | *Artibeus aequatorialis* K.Andersen, 1906 | 1 | 7 | Ec-1666; M-4111 |
| *Mastoptera guimaraesi* Wenzel, 1966 | 14 | *Phyllostomus hastatus* (Pallas, 1767) | 4 | 12 | Ec.1770-1771,1792 |
| *Megistopoda aranea*  (Coquillett, 1899) | 4 | *Artibeus aequatorialis* | 2 | 7 | Ec-1662,1665; M-4110, 4111 |
| *Megistopoda proxima* (Séguy, 1926) | 13 | *Sturnira giannae* | 10 | 4, 6, 7, 11, 13 | Ec-1621, 1650, 1667, 1685, 1695, 1697, 1765, 1766, 1768, 1793; M- 4097, 4098, 4115, 4116, 4351, 4352, 4354, 4439 |
| *Paratrichobius longicrus* (Ribeiro, 1907) | 12 | *Artibeus lituratus* (Olfers, 1818) | 4 | 5, 6, 11, 9 | Ec-1634, 1645, 1661, 1698; M-4099, 4100, 4339 |
|  | 1 | *Platyrrhinus vittatus* (Peters,1860) | 1 | 2 | Ec-075; M-1664 |
| *Speiseria ambigua* Kessel, 1925 | 6 | *Carollia perspicillata* (Linnaeus, 1758) | 6 | 5,6 | Ec-1637, 1640, 1649, 1654, 1658, 1755; M-4108, 4109, 4340 |
| *Strebla guajiro* Wenzel, 1966 | 1 | *Carollia brevicauda* (Schinz,1821) | 1 | 9 | Ec-1700, M-4340 |
|  | 1 | *Carollia perspicillata* | 1 | 9 | Ec-1702; M-4343 |
| *Strebla hertigi* Wenzel, 1966 | 1 | *Carollia brevicauda* | 1 | 9 | Ec-1759, M-4345 |
|  | 1 | *Carollia perspicillata* | 1 | 6 | Ec-1647; M-4109 |
|  | 1 | *Phyllostomus discolor* Wagner,1843 | 1 | 7 | Ec-1683 |
|  | 8 | *Phyllostomus hastatus* | 4 | 9, 12 | Ec-1760-1761, 1762, 1791; M-4331-4333, |
| *Trichobioides perspicillatus* Pessôa and Galvão, 1937 | 26 | *Phyllostomus discolor* | 9 | 7 | Ec-1664, 1669, 1671, 1674, 1675, 1676, 1678, 1680, 1682; M-4112-4114 |
| *Trichobius costalimai* Guimarães, 1938 | 27 | *Phyllostomus discolor* | 7 | 7 | Ec-1663, 1670, 1672, 1673, 1677, 1679, 1681; M-4112-4114 |
| *Trichobius dugesioides* Wenzel, 1966 | 2 | *Phyllostomus hastatus* | 2 | 9, 12 | Ec-1763, 1769; M-4334; M-4102 |
| *Trichobius joblingi* Wenzel, 1966 | 2 | *Anoura cadenai* Mantilla-Meluk and Baker, 2006 | 1 | 9 | Ec-1699; M-4355 |
|  | 14 | *Carollia brevicauda* | 8 | 8, 9, 11 | Ec-1687, 1689, 1698, 1691, 1694, 1701, 1756, 1758, 1767; M-4339-4341 |
|  | 1 | *Carollia castanea* H.Allen, 1890 | 1 | 7 | Ec-1684; M-4117 |
|  | 58 | *Carollia perspicillata* | 23 | 4, 5, 6,7, 8, 9 | Ec-1623, 1624, 1628, 1631, 1632, 1633, 1635, 1636, 1638, 1639, 1641, 1642, 1643, 1644, 1648, 1652, 1653, 1657, 1668, 1686, 1688, 1690, 1692, 1693, 1696, 1757; M-4107, 4108, 4109, 4338 |
| *Trichobius longipes* (Rudow, 1871) | 4 | *Phyllostomus hastatus* | 1 | 12 | Ec- 1772 |
| *Trichobius uniformis* Curran, 1935 | 3 | *Carollia brevicauda* | 1 | 5 | Ec-1629, 1630; M-4105 |
|  | 4 | *Glosophaga soricina* | 4 | 6 | Ec-1646, 1655, 1660; M-4096 |

**Supplementary Table 3** Species of hyperparasites reported in bat flies associated with bats in the Neotropics.

| **Bat** | **Bat fly** | **Hyperparasite** | **Country** | **Reference** |
| --- | --- | --- | --- | --- |
| **Mormoopidae** |  |  |  |  |
| *Pteronotus parnellii* (Gray, 1843) | *Trichobius yunkeri* Wenzel, 1966 | *Gloeandromyces nycteribiidarum* Thaxter, 1931 and *G. streblae* | Costa Rica | Fritz, 1983 |
| **Phyllostomidae** |  |  |  |  |
| *Anoura geoffroyi* | *Anastrebla modestini* | *Monunguis streblida* | Brazil | da Silva Reis et al. 2019 |
|  | *Anastrebla caudifera* | *Monunguis streblida* |  |  |
|  | *Trichobius dugesii complex* | *Monunguis streblida* |  |  |
|  | *Exastinion clovisi* (Pessôa and Guimarães, 1937) | *Gloeandromyces nycteribiidarum* and Laboulbeniales sp. indet. | Mexico, Brazil | Bertola et al. 2005; Haelewaters et al. 2018b |
| *Artibeus jamaicensis* | *Megistopoda aranea* | *Gloeandromyces nycteribiidarum* | Grenada, Panama | Thaxter 1931, Walker et al. 2018 |
|  |  | *Gloeandromyces streblae* and *Nycteromyces streblidinus* Thaxter, 1931 | Panamá | Walker et al. 2018 |
|  |  | *Monunguis streblida* | Mexico | Lindquist and Vercammen-Grandjean 1971 |
| *Artibeus lituratus* | *Paratrichobius longicrus* | *Laboulbeniales* sp. indet. | Brazil | Bertola et al. 2005 |
| *Carollia brevicauda* | *Trichobius joblingi* | *Gloeandromyces* sp. nov. 1 and *Nycteromyces streblidinus* | Panamá | Walker et al. 2018 |
| *Carollia castanea* | *Trichobius joblingi* | *Nycteromyces streblidinus* | Panamá | Haelewaters et al. 2018b |
| *Carollia perspicillata* | *Speiseria ambigua* | *Gloeandromyces streblae,* *Gloeandromyces* sp. nov. indet. *Nycteromyces streblidinus* and Laboulbeniales gen. & sp. indet. | Panamá, Honduras, Ecuador, Costa Rica | Haelewaters et al., 2018b; Walker et al. 2018, Fritz 1983 |
|  | *Strebla guajiro* (Garcia and Casal, 1965) | Laboulbeniales gen. & sp. indet. | Costa Rica | Fritz 1983 |
|  | *Trichobius joblingi* | ***Monunguis streblida***  *Gloeandromyces dicki* Haelew*, G. streblae, G. streblae f. sigmomorphus G.* sp. nov. 1*, G.* sp. nov. 2, *G.* sp. nov. 3*, G.* sp. nov. Indet, *G. pageanus f. alarum, G. pageanus f. polymorphus, Nycteromyces streblidinus,* Laboulbeniales sp. indet | **Colombia**, Costa Rica, Nicaragua, Panamá, Trinidad and Tobago | **This study**  Walker et al.2018; Haelewaters and Pfister 2019; Haelewaters et al. 2017b; Fritz 1983 |
| *Desmodus rotundus* (E. Geoffroy, 1810) | *Strebla wiedemanni* Kolenati, 1863 | *Gloeandromyces strebla* and *Nycteromyces streblidinus* | Venezuela | Thaxter 1917, Thaxter 1931 |
|  | *Trichobius parasiticus* Gervais, 1844 | *Nycteromyces streblidinus* | Panama | Haelewaters et al. 2018b |
| *Glossophaga soricina* | *Trichobius dugesii* | *Monunguis streblida* | Mexico | Lindquist and Vercammen-Grandjean1971 |
| *Phyllostomus discolor* | *Trichobius costalimai* | *Gloeandromyces nycteribiidarum* | Panama | Haelewaters et al 2018 |
| *Phyllostomus hastatus* | *Mastoptera guimaraesi* | *Gloeandromyces hilleri* Haelew. and Pfliegler | Ecuador, Panama | Liu et al. 2020, Haelewaters and Pfister 2019 |
|  | *Trichobius longipes* | *Gloeandromyces dicki* | Ecuador | Haelewaters and Pfister 2019 |
| ***Platyrrhinus vittatus*** | ***Paratrichobius longicrus*** | ***Gloeandromyces pageanus f. polymorphus*** | **Colombia** | **This study** |
| *Sturnira* sp. | *Megistopoda proxima* | *Laboulbeniales* sp. indet. | Brazil | Bertola et al. 2005 |
| *Trachops cirrhosus* (Spix, 1823) | *Trichobius dugesioides* | *Gloeandromyces pageanus* and *G. streblae* | Panamá | Haelewaters et al. 2017b, Walker et al. 2018 |
| **Vespertilionidae** |  |  |  |  |
| *Myotis nigricans* | *Trichobius pseudotruncatus* | *Monunguis streblida* | Dominican island | Lindquist and Vercammen-Grandjean 1971 |

**
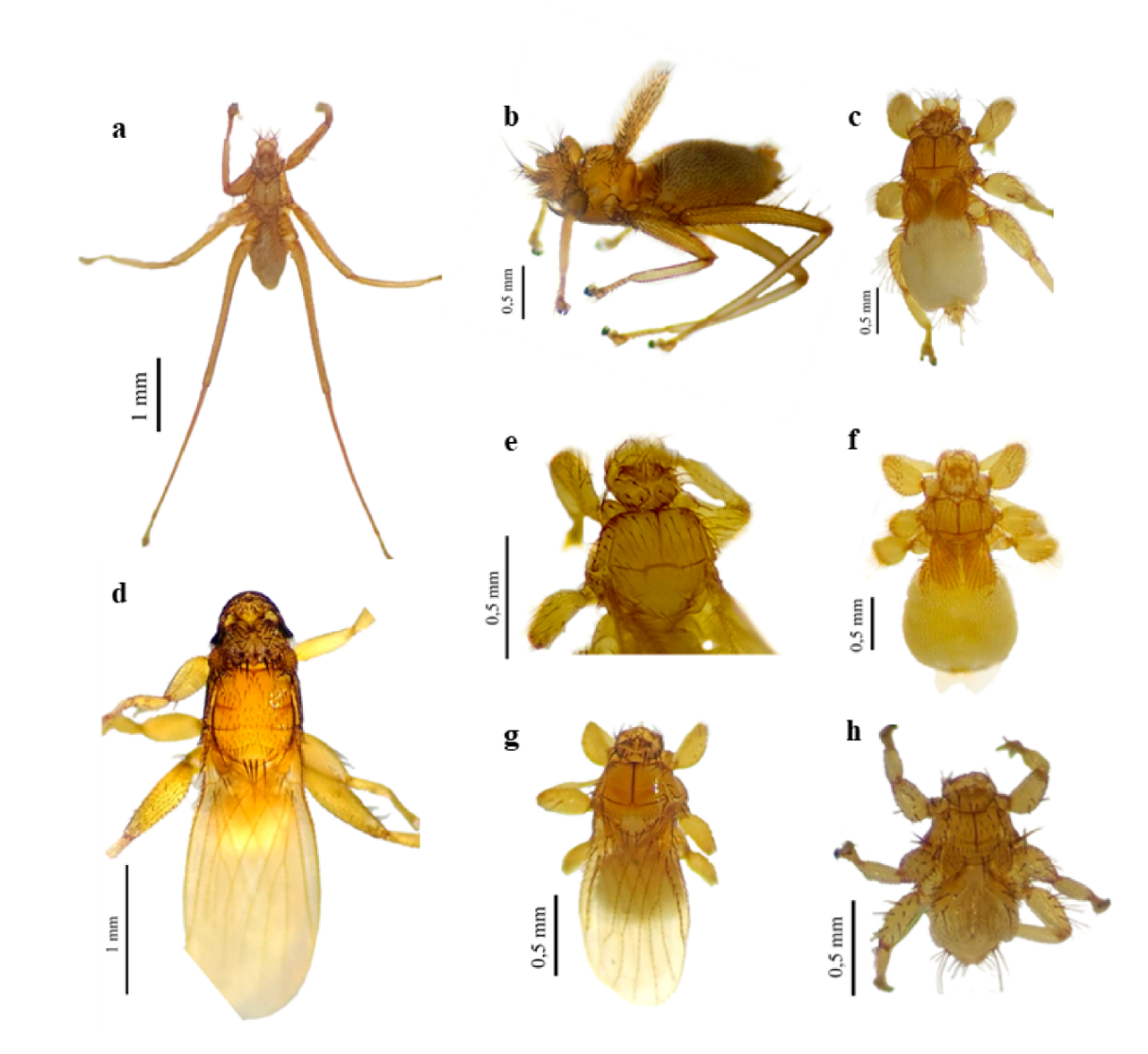
**

**Fig. S1.** Examples of bat fly species reported at the study área. **a** *Megistopoda aranea* **b** *Megistopoda proxima* **c** *Aspidoptera phyllostomatis* **d** *Strebla guajiro* **e** *Trichobius uniformis* **f** *Aspidoptera delatorrei* **g** *Trichobioides perspicillatus* **h** *Mastoptera guimaraesi*
